# Antioxidant responses and photosynthetic behaviors of *Kappaphycus alvarezii* and *Kappaphycus striatum* (Rhodophyta, Solieriaceae) during low temperature stress

**DOI:** 10.1186/s40529-016-0136-8

**Published:** 2016-08-10

**Authors:** Hu Li, Jianguo Liu, Litao Zhang, Tong Pang

**Affiliations:** 1 Key Laboratory of Experimental Marine Biology, National & Local Joint Engineering Laboratory of Ecological Mariculture, Institute of Oceanology, Chinese Academy of Sciences, 7 Nanhai Road, Qingdao, 266071 China; 2 Laboratory for Marine Biology and Biotechnology, Qingdao National Laboratory for Marine Science and Technology, 1 Wenhai Road, Aoshanwei Town, Jimo, Qingdao, 266071 China; 3 University of Chinese Academy of Sciences, Beijing, 100049 China

**Keywords:** Antioxidant systems, *Kappaphycus alvarezii*, *Kappaphycus striatum*, Low temperature stress, Photosystem II

## Abstract

**Background:**

*Kappaphycus* are farmed in tropical countries as raw material for carrageenan, which is widely used in food industry. The sea area available for farming is one limiting factor in the production of seaweeds. Though cultivation is spreading into subtropical regions, the lower seawater temperature is an important problem encountered in subtropical regions for the farming of *Kappaphycus*. This research of physiological response to low temperature stress will be helpful for screening *Kappaphycus* strains for growth in a lower temperature environment.

**Results:**

Responses of antioxidant systems and photosystem II (PSII) behaviors in *Kappaphycus alvarezii* and *Kappaphycus striatum* were evaluated during low temperature treatments (23, 20, 17 °C). Compared with the controls at 26 °C, the H_2_O_2_ concentrations increased in both species when the thalli were exposed to low temperatures (23, 20, 17 °C), but these increases were much greater in *K. striatum* than in *K. alvarezii* thalli, suggesting that *K. striatum* suffered more oxidative stress. The activities of some important antioxidant enzymes (e.g. superoxide dismutase and ascorbate peroxidase) and the hydroxyl free radical scavenging capacity were substantially higher at 23, 20 and 17 °C than at the control 26 °C in *K. alvarezii*, indicating that the antioxidant system of *K. alvarezii* enhanced its resistance to low temperature. However, no significant increases of antioxidant enzymes activities were observed at 20 and 17 °C in *K. striatum*. In addition, both the maximal efficiency of PSII photochemistry (F_V_/F_m_) and the performance index (PI_ABS_) decreased significantly in *K. striatum* at 23 °C, indicating that the photosynthetic apparatus was damaged at 23 °C. In contrast, no significant decreases of either F_V_/F_m_ or PI_ABS_ were observed in *K. alvarezii* at 23 °C.

**Conclusions:**

It is concluded that *K. alvarezii* has greater tolerance to low temperature than *K. striatum.*

## Background

*Kappaphycus alvarezii* and *Kappaphycus striatum* (Rhodophyta, Solieriaceae), two important carrageenophyte species, are used as the major commercial source of κ-carrageenan, which is widely utilized as a gelling and stabilizing agent for some food products, including frozen desserts, chocolate-flavoured milk, cottage cheese dressings and soyamilk (Doty 1973; Glenn and Doty 1990; Bixler et al. 2001; Bindu and Levine 2011). The farming of *Kappaphycus* occurs mainly in tropical southeast Asian countries (Ohno et al. 1994), such as the Philippines and Indonesia, serving as a means of livelihood for locals and as a source of revenue for the economy of these countries (Bindu 2011; Bixler and Porse 2011; Ganzon-Fortes et al. 2012). However, although the carrageenan processing technology in this industry is mature (Bixler et al. 2001), the supply of carrageenan is not adequate for the global demand. The sea area available for farming is one limiting factor in the production of seaweeds for carrageenan extraction. Currently, cultivation is spreading into subtropical regions (i.e. lower temperature regions for these seaweeds). For example, cultivation during the summer season was introduced in Shikoku Island, Southern Japan (Ohno et al. 1994). The lower seawater temperature is an important problem encountered in subtropical regions, or other low-temperature sea areas, for the farming of *Kappaphycus*.

Photosynthetic processes are exceedingly sensitive to low temperature (Allen and Ort 2001). When plants are exposed to low-temperature stress, photosynthetic enzymes may be degraded and photo-damage may occur, which would lead to decreased photosynthetic activity. The reduced photosynthesis may result in the accumulation of excess energy, which leads to the generation of numerous reactive oxygen species (ROS), such as hydrogen peroxide (H_2_O_2_) and hydroxyl free radical (·OH), which are harmful to the plant’s photosynthetic apparatus. Collén et al. (1994) found that *Kappaphycus* and *Eucheuma* exposed to abiotic or biotic stresses produced H_2_O_2_. The accumulation of H_2_O_2_ within plant tissues is thought to be a crucial event in initiating adequate antioxidant responses in plants (Gechev et al. 2003). In order to gain a better understanding of the antioxidant response of *Kappaphycus* under low temperature stress, we measured the activities of antioxidant enzymes [e.g. superoxide dismutase (SOD), catalase (CAT) and ascorbate peroxidase (APX)], hydroxyl free radical (·OH) scavenging capacity and the level of malondialdehyde (MDA), an important index of oxidative lesions (Barros et al. 2003), in *K. alvarezii* and *K. striatum* thalli under low temperature stress. Moreover, since PSII is the most sensitive part of the photosynthetic apparatus, changes of PSII state may indicate changes in physiological processes in plants (Lim et al. 2014). In recent years, the chlorophyll (Chl) a fluorescence transient (O-J-I-P) has become an important tool in photosynthesis research (Strasser et al. 2004; Strauss et al. 2007), and especially in studying PSII behaviors. The changes of the PSII antenna, the reaction centers, the oxidation–reduction of the plastoquinone (PQ) pool and the electron flow limitations on the acceptor side of PSII, can be reflected by the O-J-I-P transient modulation in higher plants and algae (Stirbet and Govindjee 2012). The effect of low temperature stress on PSII was further assessed in *K. alvarezii* and *K. striatum* thalli by means of the Chl a fluorescence transient.

## Methods

### Algal sample collection and pretreatment

**Fig. 1:**
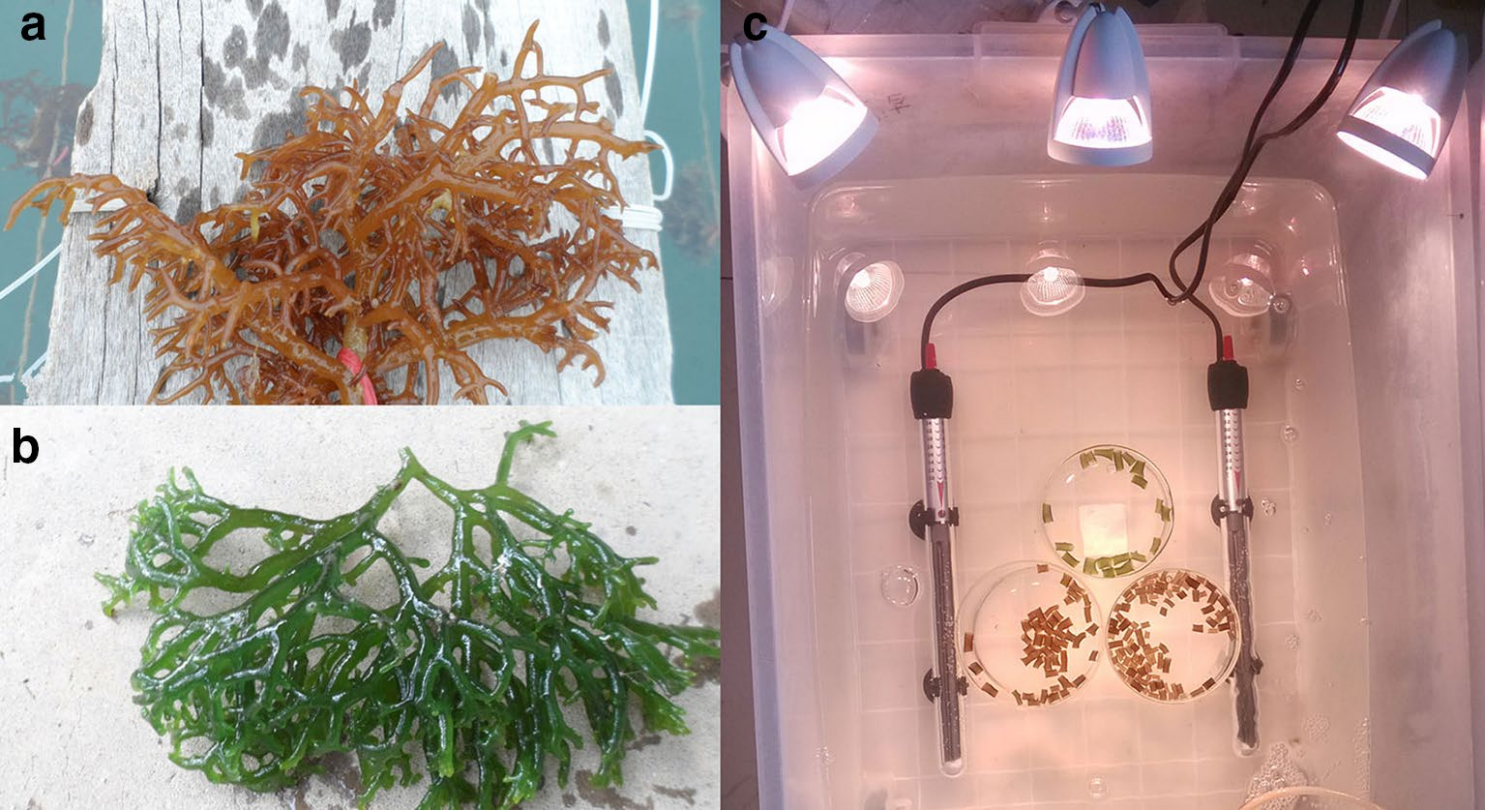
The photos of *K. alvarezii* and *K. striatum*. **a** *Kappaphycus alvarezii* thalli; **b** *Kappaphycus striatum* thalli; **c** A photo of temperature treatments of *K. alvarezii* and *K. striatum* samples in the tank. Algal samples were irradiated at 55 μmol photons m^−2^ s^−1^. The sea water temperature in the tank was adjusted and maintained by an automatic temperature-control bar

*Kappaphycus alvarezii* (Rhodophyta, Solieriaceae) (reddish brown, Fig. 1a) and *Kappaphycus striatum* (Rhodophyta, Solieriaceae) (green, Fig. 1b) were harvested in Li’an bay, Hainan Province, China (18°24′N, 110°03′E) (Liu et al. 2009; Pang et al. 2015). Selected healthy thalli of both species were cleaned to remove sludge, miscellaneous algal contaminants and other impurities from the surface of thalli. The algal thalli were acclimatized in a plastic tank (50 × 40 × 40 cm) with 40 L of natural seawater (about 23–24 °C, salinity 33 ‰) collected in Li’an bay (18°24′N, 110°03′E) and irradiated with light (55 μmol photons m^−2^ s^−1^) in a 12:12 h light:dark (L:D) cycle. They were acclimatized to their new surroundings for a day in the tank before performing the following low temperatures treatments assay.

### Low temperature treatments

Portions of thalli approximately 3 mm in diameter and 1 cm long were cut from the apices of the collected algal thalli. The samples were taken from same parts of the thallus to avoid any difference between individuals. These were all placed in plastic tanks (50 × 40 × 40 cm) with 40 L of natural seawater (about 23–24 °C, salinity 33 ‰) collected in Li’an bay (18°24′N, 110°03′E) and irradiated with a fluorescent lamp at approximately 55 μmol photons m^−2^ s^−1^ (Fig. 1c). Sea temperature was adjusted and maintained by an automatic temperature control unit (Guangdong Zhenhua Electrical Appliance Co., LTD). The control temperature was 26 °C, which is the optimum temperature for these two species (Lideman et al. 2013). The samples of two species were exposed to the control temperature (26 °C) and low temperatures 23, 20 and 17 °C for 2 h, respectively. The temperature treatments of *K. alvarezii* and *K. striatum* samples were shown in Fig. 1c.

### Crude extracts and antioxidant system parameters

After treatment at each temperature, algal samples of each species were immediately froze in liquid nitrogen, labeled and stored in a freezer at −80 °C for the following assay (Fariduddin et al. 2014).

The frozen samples (about 2 g) were ground in a mortar in liquid nitrogen and placed in 10-mL centrifuge tubes in ice. The algal powder was mixed with 3 mL of pre-cooled extraction buffer (50 mM phosphate buffer, pH 7.2, 0.1 mM EDTA) and homogenized using an S10 homogenizer (Ningbo Xinzhi Biotechnology Co., Ltd) in an ice-water bath (Nagarani and Kumaraguru 2012). Cell debris was removed by centrifugation at 12,000*g* for 10 min at 4 °C and the supernatant was used for the following measurements of antioxidant system parameters. Each temperature treatment had three repeats for each species.

The concentration of H_2_O_2_ in the algal thalli was assessed using a commercially available kit—Hydrogen Peroxide Assay kit (Nanjing Jiancheng Bioengineering Institute). H_2_O_2_ was bound with molybdenic acid to form a complex, which was measured spectrophotometrically at 405 nm and the concentration of H_2_O_2_ was then calculated (Liu et al. 2013).

Total SOD activity in algal thalli was determined by using a Total Superoxide Dismutase Assay kit (Nanjing Jiancheng Bioengineering Institute) based on measuring the enzyme’s ability to inhibit the photochemical reduction of tetrazolium blue. The developed blue color was measured spectrophotometrically at 550 nm (Das et al. 2000). CAT activity was quantified by the method of Malanga et al. (1999). The absorbance decay of H_2_O_2_ was monitored by spectrophotometry at 240 nm, with a molar extinction coefficient of ε = 39.4 mM^−1^ cm^−1^. APX activity was measured as described by Nakano and Asada (1981). Ascorbate consumption was monitored by spectrophotometry at 290 nm, with a molar extinction coefficient of ε = 2.8 mM^−1^ cm^−1^.

Hydroxyl free radical scavenging capacity (HFRSC) was determined by using a commercially available kit—Hydroxyl Free Radical Assay kit (Nanjing Jiancheng Bioengineering Institute) based on the deoxyribose degradation assay described by Halliwell et al. (2006).

To evaluate the extent of oxidative damage in algal cells under low temperature stress, the concentration of MDA, a marker of lipoperoxidation, was measured in the algal thalli by the method of Fraga et al. (1988).

### Protein determination

Enzyme activities were expressed relative to protein concentrations, which were estimated by the method of Deng et al. (2013) using bovine serum albumin as standard. We employed the dying method of Coomassie brilliant blue G-250 to determine the protein concentration of algal samples, measuring the absorbency at the wavelength of 595 nm. Each temperature treatment had three repeats for each species.

### Chlorophyll (Chl) a fluorescence transient measurement

Fast Chla fluorescence transients were measured with a Plant Efficiency Analyzer (Handy-PEA, Hansatech Instruments Ltd, UK). Each transient obtained from the samples was analyzed according to the JIP-test (Strasser 1978; Strasser et al. 2004; Stirbet and Govindjee 2012; Zhang et al. 2012a). Each temperature treatment had 10 repeats for each species.

The following variables were calculated directly or indirectly.

1. $\upvarphi_{\text{Po}}$ (or F_V_/F_m_), the maximum PSII photochemical efficiency, namely the maximum quantum yield of primary photochemistry. F_V_/F_m_ = $\upvarphi_{\text{Po}}$ = TR_O_/ABS, where TR_O_ and ABS denote the trapped and absorbed excitation energy fluxes, respectively.
2. ψ_o_, the probability that a trapped exciton moved an electron into the electron transport chain beyond (plasto)quinone (Q_A_^−^). ψ_o_ = ET_O_/TR_O_, where ET_O_ and TR_O_ denote the electron transport and trapped excitation energy fluxes, respectively.
3. $\upvarphi_{\text{Eo}}$, the probability that an absorbed photon moved an electron into the electron transport chain further than Q_A_^−^, namely quantum yield of electron transport. $\upvarphi_{\text{Eo}}$ = ET_O_/ABS.
4. PI_ABS_, the performance index on an absorption basis. PI_ABS_ = (RC/ABS)·[$\upvarphi_{\text{Po}}$/(1 −$\upvarphi_{\text{Po}}$)]·[ψ_o_/(1 − ψ_o_)], where RC/ABS, $\upvarphi_{\text{Po}}$/(1 − $\upvarphi_{\text{Po}}$) and ψ_o_/(1 − ψ_o_) reflect the efficiency of the reaction centers, the light energy absorption efficiency and the accepting efficiency of the electron acceptor, respectively.

### Statistical analysis

Data are presented as mean ± SD for at least three replicate measurements (the number (n) of replicates is presented in each figure caption). To determine the statistical significance between experimental groups, the data were examined using a *t* test (p < 0.05).

## Results

### Changes in H_2_O_2_ concentrations in *Kappaphycus alvarezii* and *Kappaphycus striatum* thalli

**Fig. 2:**
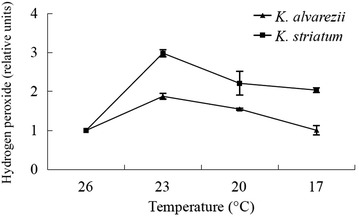
Concentrations of H_2_O_2_ in thalli of *K. alvarezii* and *K. striatum* exposed to low temperatures (23, 20 and 17 °C) for 2 h, relative to the concentration of the control of each species at 26 °C [H_2_O_2_ concentration in thalli at the control temperature (26 °C) was 17.7 ± 1.3 mmol g^−1^ prot for *K. alvarezii* and 29.2 ± 2.0 mmol g^−1^ prot for *K. striatum*]. Data are means ± SD of three replicates (n = 3)

The highest concentrations of H_2_O_2_ in both *K. alvarezii* and *K. striatum* thalli were observed at 23 °C; they decreased at 20 and 17 °C. A H_2_O_2_ concentration similar to the control (26 °C) was observed at 17 °C in *K. alvarezii* thalli but *K. striatum* thalli at 17 °C still had a higher H_2_O_2_ concentration than the control at 26 °C (Fig. 2). In other words, compared with the controls at 26 °C, the H_2_O_2_ concentration increased in both species when the thalli were exposed to low temperatures (23, 20, 17 °C), but the increase was much greater in *K. striatum* thalli than in *K. alvarezii* thalli. For example, the H_2_O_2_ concentration at 23 °C was 1.9 times as much as that of the control in *K. alvarezii* thalli, while the H_2_O_2_ concentration at 23 °C was 3 times as much as that of the control in *K. striatum* thalli. These results suggest that *K. striatum* thalli suffered more serious oxidative stress from H_2_O_2_ than *K. alvarezii* thalli.

### Antioxidant system responses of *K. alvarezii* and *K. striatum* thalli

**Fig. 3:**
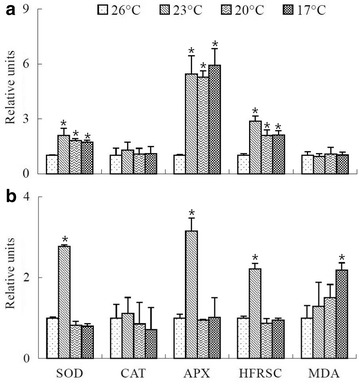
Superoxide dismutase (SOD), catalase (CAT), and ascorbate peroxidase (APX) activities and hydroxyl free radical scavenging capacity (HFRSC) and concentrations of malonaldehyde (MDA) in *Kappaphycus alvarezii* (**a**) and *Kappaphycus striatum* (**b**) thalli exposed to 26 °C (the control) and low temperatures (23, 20 and 17 °C) for 2 h, respectively. All values of the above parameters are expressed relative to those of their controls (the controls are taken as 1) and are presented in relative units. (Data are means ± SD of three replicates (n = 3). For each parameter, for each species, the treatments with a* star* (*) are significantly different from the respective control, p < 0.05)

Low temperature treatments resulted in distinct responses from the three major antioxidant enzymes (SOD, APX and CAT) in both *K. alvarezii* and *K. striatum* thalli. Compared with the control, SOD activities and APX activities increased significantly in both species when algal thalli were exposed to 23 °C (SOD activities at 23 °C were 2.1 times and 2.8 times as much as that of the controls in *K. alvarezii* thalli and *K. striatum* thalli, respectively; APX activities at 23 °C were 5.5 times and 3.1 times as much as that of the controls in *K. alvarezii* thalli and *K. striatum* thalli, respectively) (Fig. 3). At even lower temperatures (20 and 17 °C), SOD activities and APX activities of *K. alvarezii* thalli remained elevated (SOD activities were 1.8 times and 1.7 times as much as that of the control, at 20 and 17 °C, respectively; APX activities were 5.3 times and 5.9 times as much as that of the control, at 20 and 17 °C, respectively), whereas those of *K. striatum* thalli had no significant differences at either 20 or 17 °C compared with the control. Although CAT activities did not vary significantly in the two species after exposure to low temperatures (23, 20 and 17 °C) (Fig. 3), *K. alvarezii* thalli maintained higher SOD and APX activities whereas *K. striatum* thalli didn’t, under the low temperatures (20 and 17 °C). In addition, compared with the control, hydroxyl free radical scavenging capacity (HFRSC) had significant increases at lowered temperatures (HFRSC were 2.9 times, 2.1 times and 2.1 times as much as the control, at 23, 20 and 17 °C, respectively) in *K. alvarezii* thalli. Hydroxyl free radical scavenging capacity (HFRSC) of *K. striatum* thalli peaked at 23 °C (HFRSC was 2.2 times as much as the control), but dropped to the same level as the control at the lower temperatures, 20 and 17 °C (Fig. 3). Taken together, these results suggest that the antioxidant system of these species increased their activities against the oxidative stress generated by exposure to a low temperature of 23 °C. The antioxidant system of *K. striatum* thalli was unable to resist the lower temperatures (20 and 17 °C) whereas that of *K. alvarezii* thalli still had resistance to the lower temperatures (20 and 17 °C). No significant differences of MDA were found in the two species during exposure to lower temperatures (23 and 20 °C) (Fig. 3). At the lowest temperature of 17 °C, a significant increase of MDA (MDA concentration at 17 °C was 2.2 times as much as the control) was observed in *K. striatum* thalli, which confirmed the occurrence of oxidative damage; however, no significant increase of MDA was observed in *K. alvarezii* thalli (Fig. 3).

### Changes in photosynthetic efficiencies (or energy flux ratios)—$\upvarphi_{\text{Po}}$, ψ_o_ and $\upvarphi_{\text{Eo}}$

**Fig. 4:**
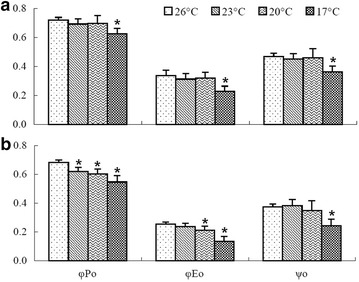
Energy flux ratios ($\upvarphi_{\text{Po}}$, $\upvarphi_{\text{Eo}}$ and ψ_o_) of *Kappaphycus alvarezii* (**a**) and *Kappaphycus striatum* (**b**) thalli after exposure to 26 °C (the control) and low temperatures (23, 20 and 17 °C) for 2 h, respectively. (Means ± SD of ten replicates (n = 10) are presented. For each species, the treatment with a* star* (*) indicates that there is a significant difference between this temperature treatment and the control (26 °C), p < 0.05)

Compared with the control, a significant decrease in the maximum PSII photochemical efficiency (F_V_/F_m_ = $\upvarphi_{\text{Po}}$) was observed in *K. striatum* thalli at 23 °C (the F_V_/F_m_ value was 0.62 while the control was 0.68) (Fig. 4), indicating that photo-damage had occurred. However, in *K. alvarezii* thalli, there was no significant decrease of F_V_/F_m_ at that temperature (23 °C). When exposed to 17 °C, both *K. alvarezii* and *K. striatum* thalli had significant decreases in F_V_/F_m_ compared to the control (F_V_/F_m_ values were 0.63 and 0.55 at 17 °C in *K. alvarezii* and *K. striatum* thalli, respectively, whereas the respective controls were 0.72 and 0.68). Because the value of F_V_/F_m_ largely reflected the physiological activity in plants, it was inferred that physiological activity in *K. alvarezii* might have greater tolerance to low temperature stress than that in *K. striatum*.

With regard to the excitation efficiency for electron transport beyond Q_A_^−^ (ψ_o_), both *K. alvarezii* and *K. striatum* thalli only exhibited significant decreases of ψ_o_ when the temperature dropped to 17 °C (ψ_o_ values were 0.36 and 0.24 in *K. alvarezii* and *K. striatum* thalli, respectively, whereas the respective controls were 0.47 and 0.37) (Fig. 4). At 23 and 20 °C, no significant decreases of ψ_o_ were observed, indicating that the electron transport in the PSII electron transport chain was not influenced significantly in either species. Compared with the control, the quantum yield of electron transport ($\upvarphi_{\text{Eo}}$) in *K. striatum* thalli decreased significantly at 20 and 17 °C ($\upvarphi_{\text{Eo}}$ values were 0.21 and 0.13 at 20 and 17 °C, respectively, while the control was 0.26) (Fig. 4) while a significant decrease of $\upvarphi_{\text{Eo}}$ was only observed at 17 °C ($\upvarphi_{\text{Eo}}$ value was 0.23 at 17 °C while the control was 0.34) in *K. alvarezii* thalli. These results suggest that the lowered temperature of 20 °C led to a decreased quantum yield of electron transport in *K. striatum* thalli, whereas *K. alvarezii* thalli maintained a normal capacity for electron transport at that same temperature (20 °C), indicating again that *K. alvarezii* had stronger low temperature tolerance than *K. striatum* with regard to electron transport.

Since the parameters of photosynthetic efficiencies only reflect the energy cascade from light absorption to electron transport and don’t indicate the specific PSII component damaged by low temperature stress, the performance index on an absorption basis (PI_ABS_) and its three variables RC/ABS, $\upvarphi_{\text{Po}}$/(1 − $\upvarphi_{\text{Po}}$), ψo/(1 − ψo), were calculated to probe the specific component of PSII possibly damaged by low temperature stress.

### Changes in the performance index—PI_ABS_

**Fig. 5:**
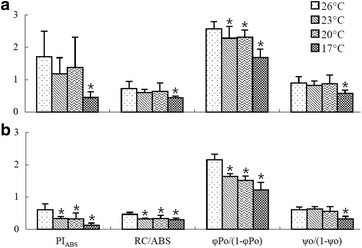
Performance index on an absorption basis (PI_ABS_) as well as its variables RC/ABS, $\upvarphi_{\text{Po}}$/(1 − $\upvarphi_{\text{Po}}$) and ψo/(1 − ψo) in *Kappaphycus alvarezii* (**a**) and *Kappaphycus striatum* (**b**) thalli after exposure to 26 °C (the control) and low temperatures (23, 20 and 17 °C) for 2 h, respectively. (Data are means ± SD of ten replicates (n = 10). For each parameter, for each species, the treatments with a star (*) are significantly different from the respective control (26 °C), p < 0.05)

PI_ABS_ is more sensitive to changes of photosynthetic activity than the maximal photochemical efficiency (F_V_/F_m_). Compared with the control, there were significant decreases of PI_ABS_ in *K. striatum* thalli when the temperature was below 23 °C (PI_ABS_ values were 0.33, 0.32 and 0.12 at 23, 20 and 17 °C, respectively, while the control was 0.61), whereas a significant decrease of PI_ABS_ was observed in *K. alvarezii* thalli only when the temperature was dropped to 17 °C (PI_ABS_ value was 0.45 at 17 °C while the control was 1.71) (Fig. 5). This result suggests that the 3 °C decrement in temperature from 26 °C resulted in decreased photosynthetic activity of PSII in *K. striatum* thalli, whereas a 9 °C decrement from 26 °C was needed for a negative impact on the PSII activity in *K. alvarezii* thalli.

In order to explore responses of different parts of PSII in the two species during the low temperature stress, three variables RC/ABS, $\upvarphi_{\text{Po}}$/(1 −$\upvarphi_{\text{Po}}$) and ψ_o_/(1 − ψ_o_), were calculated. RC/ABS, $\upvarphi_{\text{Po}}$/(1 − $\upvarphi_{\text{Po}}$) and ψ_o_/(1 − ψ_o_) reflect the efficiency of reaction centers, the light energy absorption efficiency and the accepting efficiency of the electron acceptor, respectively. When the temperature was dropped to 23 °C, RC/ABS decreased significantly in *K. striatum* thalli (the ratio RC/ABS was 0.32 at 23 °C while the control was 0.46), whereas no significant decrease of RC/ABS was observed in *K. alvarezii* until the temperature was decreased to 17 °C (the ratio RC/ABS was 0.45 at 17 °C while the control was 0.72) (Fig. 5). This suggests that reaction centers of PSII in *K. alvarezii* thalli maintained normal efficiency at 23 and 20 °C whereas the efficiency of PSII reaction centers in *K. striatum* thalli had decreased at these temperatures. At 17 °C, PSII reaction centers of both *K. alvarezii* and *K. striatum* were damaged. However, both *K. alvarezii* and *K. striatum* thalli had significant decreases of $\upvarphi_{\text{Po}}$/(1 −$\upvarphi_{\text{Po}}$) at 23 °C (the ratios $\upvarphi_{\text{Po}}$/(1 − $\upvarphi_{\text{Po}}$) were 2.2 and 1.6 in *K. alvarezii* and *K. striatum* thalli, respectively, while the respective controls were 2.6 and 2.2), indicating that the low temperature reduced the efficiency of the PSII antenna in both species (Fig. 5). In addition, the accepting efficiencies of the PSII electron acceptor (ψ_o_/(1 − ψ_o_)) in both *K. alvarezii* thalli and *K. striatum* thalli were impacted negatively at 17 °C (the ratios ψ_o_/(1 − ψ_o_) were 0.58 and 0.32 in *K. alvarezii* and *K. striatum* thalli, respectively, while the respective controls were 0.90 and 0.60), indicating that the acceptor sides of PSII in these two species were damaged (Fig. 5). In summary, in both species, PSII antennae were first damaged by the low temperature stress; damages to the PSII acceptor side was observed only at the lowest temperature (17 °C). In addition, the PSII reaction centers of *K. striatum* had a lower resistance to the low temperature stress than those of *K. alvarezii.*

## Discussion

PSII contains some sensitive proteins, and the state of PSII is easily affected by low temperature stress (Čajánek et al. 1998; Tang et al. 2007). After exposure to low temperature stress, physiological dysfunctions, including alteration of metabolic processes, increase in ROS and reduction of photosynthetic capacity occur in plants (Allen and Ort 2001). The harsh conditions imposed by various environmental stresses result in an increase of H_2_O_2_, which is the main ROS in *K. alvarezii* thalli (Reis et al. 2011; Ling et al. 2015). Moreover, Barros et al. (2006) found that when *K. alvarezii* thalli were subjected to two co-stressors, chilling and high light, the H_2_O_2_ level increased. In our study, increases of H_2_O_2_ levels (Fig. 2) were observed during exposure to lowered temperatures (23 and 20 °C) in both *K. alvarezii* and *K. striatum* thalli, suggesting that oxidative stress occurred, but this increases were much greater in *K. striatum* thalli than in *K. alvarezii* thalli, indicating that *K. striatum* suffered more serious oxidative stress from H_2_O_2_ than *K. alvarezii*. *Kappaphycus alvarezii* at 17 °C had a similar H_2_O_2_ level as the control at 26°. That might be due to the increase of H_2_O_2_—scavenging enzyme (such as APX) activities. Barros et al. (2003) found that *K. alvarezii* released H_2_O_2_ into the surrounding medium to avoid harmful accumulation of H_2_O_2_. But *K. striatum* at 17 °C still had a higher H_2_O_2_ level than the control at 26°. Above results indicated that *K. striatum* might suffer greater oxidative damage than *K. alvarezii* during the low temperature.

Some important antioxidant enzymes (SOD, APX and CAT) of *K. alvarezii* increased their activities when *K. alvarezii* thalli is exposed to the cold and high light stress (Barros et al. 2006). In our study, SOD and APX activities were substantially higher at 23 °C than at 26 °C (Fig. 3) in both two species, indicating the antioxidant systems of both species enhanced their antioxidant activities against the oxidative stress caused by the low temperature. However, compared with the control, no significant increases of SOD and APX activities in *K. striatum* thalli were observed at the lower temperatures of 20 and 17 °C, whereas SOD and APX activities of *K. alvarezii* thalli were still higher at these lower temperatures (Fig. 3), indicating that these two antioxidant enzymes of *K. alvarezii* thalli persisted at the lower temperatures (20 and 17 °C) but those of *K. striatum* thalli didn’t. However, it is worth noting that no significant difference of CAT activity was observed during any of the low temperature treatments in either of the two species. Because CAT and APX are two important H_2_O_2_-scavenging enzymes, why doesn’t CAT respond to the increase of H_2_O_2_? Barros et al. (2003) found that APX was an effective system for decomposing lower concentrations of H_2_O_2_ in *K. alvarezii*, while higher concentrations of H_2_O_2_ woud be more efficiently decomposed by CAT. Ascorbate peroxidase (APX) has a greater affinity for H_2_O_2_ than CAT. So it is surmised that concentrations of H_2_O_2_ didn’t reach the level at which CAT would play a role during these low temperature treatments. Not only H_2_O_2_, but also ·OH (a powerful oxidizing agent) can cross membranes and may oxidize a variety of compounds (Nagarani and Kumaraguru 2012). Hydroxyl free radical scavenging capacity (HFRSC) was congruent with SOD and APX activities for each species (Fig. 3). A significant increase of HFRSC persisted in *K. alvarezii* thalli, whereas no significant increase of HFRSC was observed in *K. striatum* thalli at the lower temperatures (20 and 17 °C), indicating that *K. alvarezii* retained a stronger capacity for eliminating ·OH at 20 and 17 °C but *K. striatum* did not. Besides, the lipoperoxidation damage was only observed in *K. striatum* thalli (MDA, a marker of lipoperoxidation, increased significantly at 17 °C in that species) (Fig. 3). From the different antioxidant responses of these species to low temperature stress, it was inferred that *K. alvarezii* thalli might have greater low temperature resistance than *K. striatum* thalli.

Given that PSII is the most sensitive part of the photosynthetic apparatus, changes of the PSII state can indicate changes in physiological processes during low temperature stress (Lim et al. 2014). The maximum PSII photochemical efficiency decreased (F_V_/F_m_ values decreased) in both species as a consequence of low temperature stress, indicating that photo-damage had occurred such that photosynthetic activities decreased in both species.

The energy flux ratios ($\upvarphi_{\text{Po}}$, ψ_o_ and $\upvarphi_{\text{Eo}}$), which were measured by Chl a fluorescence transients, can reflect the energy utilization from light absorption to electron transport and indicate the photosynthetic efficiencies during the energy cascade (Strasser et al. 2004). Li et al. (2014) found that the maximum quantum yield for primary photochemistry ($\upvarphi_{\text{Po}}$), the efficiency at which a trapped exciton moved an electron into the electron transport chain beyond Q_A_^−^ (ψ_o_) and the quantum yield of electron transport ($\upvarphi_{\text{Eo}}$), all decreased sharply under temperature stress. In our results, a significant decrease of $\upvarphi_{\text{Po}}$ was observed in *K. striatum* thalli when the temperature dropped to 23 °C, whereas there was no significant decrease of $\upvarphi_{\text{Po}}$ at that temperature in *K. alvarezii* thalli (Fig. 4). On the other hand, $\upvarphi_{\text{Eo}}$ decreased sharply at 20 °C in *K. striatum* thalli while no significant decrease of $\upvarphi_{\text{Eo}}$ was observed in *K. alvarezii* thalli (Fig. 4), indicating electron transport of *K. alvarezii* PSII wasn’t affected at this temperature. These results suggest that *K. alvarezii* thalli had greater low temperature tolerance than *K. striatum* thalli wtih regard to the PSII energy utilization.

PI_ABS_ is more sensitive to changes in photosynthetic activity than the maximal photochemical efficiency (F_V_/F_m_) and can reflect the states of different photosynthetic components (Strasser et al. 2004). Strauss et al. (2007) found that low soil temperature decreased the value of PI_ABS_ seriously in soybeans. For red seaweeds, Zhang et al. (2012b) studied the Chl fluorescence characteristics of different strains of *Porphyra yezoensis* under low temperature stress. They found that the strains Sulian and WT had higher photochemical efficiency of PSII than the strain Sutong under low temperature stress, indicating that Sulian and WT might have better tolerance to low temperature. In our study, significant decreases of PI_ABS_ were observed in *K. striatum* thalli at 23, 20 and 17 °C, whereas a significant decrease of PI_ABS_ was observed in *K. alvarezii* thalli only at 17 °C. It indicated that the PSII of *K. alvarezii* thalli might have greater low temperature tolerance than the PSII of *K. striatum* thalli. In order to determine the low temperature tolerance of individual photosynthetic components of PSII (reaction centers, antennae and acceptor sides of PSII) for the two species, the values of RC/ABS, $\upvarphi_{\text{Po}}$/(1 − $\upvarphi_{\text{Po}}$), ψo/(1 − ψo), were measured (Fig. 5). From our results, PSII antennae were first damaged by the low temperature stress and damage of the PSII acceptor side was observed only at 17 °C in both species. RC/ABS decreased significantly in *K. striatum* thalli at 23 and 20 °C while no significant difference of RC/ABS was observed in *K. alvarezii* thalli at these temperatures. This suggests that efficiency of PSII reaction centres in *K. alvarezii* thalli remained at a normal level while the efficiency of PSII reaction centers in *K. striatum* thalli decreased at lower temperatures (23 and 20 °C). It was inferred that PSII reaction centers of *K. striatum* might have weaker resistance to low temperature stress than those of *K. alvarezii*.

## Conclusions

κ-Carrageenan-producing *K. alvarezii* and *K. striatum* are important commercial red seaweeds. Strains of *Kappaphycus* could be selected for temperature tolerance by using the Chl fluorescence method, which is non-destructive to the algal tissue and takes only a few secs to record (Zhang et al. 2012b). This study has shown that the excess H_2_O_2_ generated by low temperature stress can indirectly damage the PSII apparatus and these two species have somewhat different physiological responses to low temperature stress. By comparing the several important parameters of the Chl fluorescence transient, it is concluded that the PSII of *K. alvarezii* has a stronger low temperature tolerance than *K. striatum.* Given that photosynthesis has been considered to be the physiological process most sensitive to temperature damage and that PSII is the most sensitive part of the photosynthetic apparatus (Wen et al. 2005), it is inferred that *K. alvarezii* thalli might have stronger low temperature tolerance than *K. striatum* thalli as a consequence of PSII characteristics. Our results will be helpful for further studies on the low temperature tolerance of *Kappaphycus* and for screening *Kappaphycus* strains for growth in a lower temperature environment (or higher latitude regions).

## Abbreviations

APX: ascorbate peroxidase
CAT: catalase
Chl: chlorophyll
HFRSC: hydroxyl free radical scavenging capacity
H_2_O_2_: hydrogen peroxide
MDA: malondialdehyde
OEC: oxygen-evolving complex
·OH: hydroxyl free radical
PQ: plastoquinone
PSII: photosystem II
ROS: reactive oxygen species
SOD: superoxide dismutase
